# The clinical impact of observer variability in lung nodule classification in children with Wilms tumour

**DOI:** 10.1002/pbc.29759

**Published:** 2022-06-02

**Authors:** Jesper Sune Brok, Susan Shelmerdine, Frederikke Damsgaard, Anne Smets, Sabine Irtan, Sophie Swinson, Venus Hedayati, Joseph Jacob, Arjun Nair, Minou Oostveen, Kathy Pritchard-Jones, Øystein Olsen

**Affiliations:** 1 Department of Paediatric Haematology and Oncology, Rigshospitalet, Copenhagen University Hospital, Copenhagen, Denmark; 2 Department of Clinical Radiology, Great Ormond Street Hospital for Children NHS foundation Trust, London, UK; 3 Department of Radiology, Amsterdam University Medical Centre, Amsterdam, The Netherlands; 4 Department of Visceral and Neonatal Paediatric Surgery, Sorbonne University, Armand Trousseau Hospital - APHP, Paris, France; 5 The Leeds Teaching Hospitals, Leeds, UK; 6 King’s College Hospital NHS Foundation Trust, London, UK; 7 Centre for Medical Image Computing, University College London, London, UK; 8 Department of Respiratory Medicine, University College London, London, UK; 9 University College London Hospitals NHS Foundation Trust, London, UK; 10 UCL Great Ormond Street Institute of Child Health, London, UK

**Keywords:** child, observer variation, secondary lung neoplasms, Wilms tumour, X-ray computed tomography

## Abstract

**Objectives:**

To investigate the extent to which observer variability of computed tomography (CT) lung nodule assessment may affect clinical treatment stratification in Wilms tumour (WT) patients, according to the recent Société Internationale d’Oncologie Pédiatrique Renal Tumour Study Group (SIOP-RTSG) UMBRELLA protocol.

**Methods:**

I: CT thoraces of children with WT submitted for central review were used to estimate size distribution of lung metastases. II: Scans were selected for blinded review by five radiologists to determine intra- and inter-observer variability. They assessed identical scans on two occasions 6 months apart. III: Monte Carlo simulation (MCMC) was used to predict the clinical impact of observer variation when applying the UMBRELLA protocol size criteria.

**Results:**

Lung nodules were found in 84 out of 360 (23%) children with WT. For 21 identified lung nodules, inter-observer limits of agreement (LOA) for the five readers were ±2.4 and ±1.4 mm (AP diameter), ±1.9 and ±1.8 mm (TS diameter) and ±2.0 and ±2.4 mm (LS diameter) at assessments 1 and 2. Intra-observer LOA across the three dimensions were ±1.5, ±2.2, ±3.5, ±3.1 and ±2.6 mm (readers 1–5). MCMC demonstrated that 17% of the patients with a ‘true’ nodule size of ≥3 mm will be scored as <3 mm, and 21% of the patients with a ‘true’ nodule size of <3 mm will be scored as being ≥3 mm.

**Conclusion:**

A significant intra-inter observer variation was found when measuring lung nodules on CT for patients with WT. This may have significant implications on treatment stratification, and thereby outcome, when applying a threshold of ≥3 mm fora lung nodule to dictate metastatic status.

## 1 Background and AIM

Nearly 1000 children are diagnosed with a malignant renal tumour each year across Europe. Of these, the commonest type is Wilms tumour (WT), typically affecting children aged 2–4 years.^1^ The overall survival rate for WT is about 90% if optimal treatment is available.^2^ However, metastatic WT (stage IV), diagnosed in about 15% of the WT patients3,4 has a poorer prognosis and requires a more intensive treatment regime, which could lead to higher risks for treatment-related late effects.^5–8^

The lungs are the most common site for haematogenous dissemination of WT metastases. Lung metastases can be detected on chest X-ray (CXR) and computed tomography (CT). In the previous international protocol (SIOP 2001) from the *Société Internationale d’Oncologie Pédia-trique Renal Tumour Study Group* (SIOP-RTSG), CXR was the mandatory modality to evaluate the presence of lung metastases, and a lesion of 10 mm or greater on a high-quality CXR was considered a ‘true metastasis’. If CT was performed and lung nodules were detected on CT but not seen on CXR (i.e., ‘CT-only’ lesions too small or hidden on CXR), the protocol stated patients were to be treated preoperatively as localised disease.^9^

With CT becoming more available and easier to use on children and with the increasing awareness that nodules can be missed on CXR when situated in certain locations, there has been a gradual shift from CXR to CT as the standard imaging modality. CT provides better sensitivity for the detection of small lung nodules, but does incur a higher radiation dosage compared to CXR.

In the current SIOP-RTSG UMBRELLA protocol, chest CT is the mandatory modality for the detection of lung metastases at diagnosis.^10^ Accordingly, patients with CT-only lesions are identified (typically 3–10 mm in diameter), as they seem to have poorer outcomes compared to patients without such nodules.^6^ If lung nodules are detected, excluding clearly benign-looking nodules (e.g., granuloma and atelectasis), staging is dictated according to lung nodule size. A patient with a lung nodule of 3 mm or more in any diameter is considered to have metastatic disease and should receive the more intensive chemotherapy regimen.^9–12^ While these new staging criteria provide more specific guidance on the cut-off diameters for metastatic pulmonary disease, they also bring challenges—first that there is limited evidence for size to be a useful discriminator for malignant lung nodules and second that inter- and intra-observer variability regarding measurements may lead to incorrect categorisation of lung nodules.^10,13–15^ Such observer variability may have a great clinical impact when applying the exact size criteria for lung metastases in the UMBRELLA protocol.^10^ Variability is an inevitable part of measuring lesions on images, but the level and impact of such variability is important to quantify, and minimise, if possible.

In this study, our aims are to investigate the extent to which inter- and intra-observer variability of CT lung nodule assessment may affect clinical treatment stratification, when applying the new SIOP-RTSG UMBRELLA staging criteria for children with WT.

## 2 Methods

This study consists of three complementary analytical methods applied to chest CT imaging and their associated data from children with WT treated in the United Kingdom.

### 2.1 Method I: Central radiology review to estimate size distribution of lung nodules

From 2012 and onwards, most children in the United Kingdom and Republic of Ireland diagnosed with a renal tumour (predominantly Wilms) were enrolled in the Children’s Cancer and Leukaemia Group ‘Improving Population Outcomes of Renal Tumours of childhood’ (IMPORT) study. This is a prospective clinical observational study that aims to evaluate molecular prognostic biomarkers, and which introduced a national central radiology review process. The IMPORT study has now been incorporated into the international SIOP-RTSG UMBRELLA study.

Eligible IMPORT imaging studies (i.e., diagnostic, preoperative and relapse magnetic resonance imaging and/or CT imaging of thorax and abdomen) are centrally reviewed and subsequently stored in a research imaging library.

From 2013 to 2017, two authors (Oystein Olsen/Sabine Irtan or Oystein Olsen/Jesper Sune Brok) sitting together had reviewed imaging studies from IMPORT (both with and without knowledge of local assessment) and returned a clinical letter to the local radiologists and treating physician to take into consideration. The number and size of lung nodules, excluding clearly benign nodules (e.g., granuloma and atelectasis), were measured and recorded. From these reports, measurements were used to estimate the size distribution of the largest nodule among WT patients, if present. The measurements from this central review were used to simulate the distribution of lung nodules.

### 2.2 Method II: Blinded repeated expert review of CT thoraces to estimate observer variability

Of the chest CT studies that contained at least one lung nodule (as determined by method I above), a subset was selected for testing radiologists’ inter- and intra-observer variability. The criteria that led to select this subset was their lack of motion artefact in the image, thin slice acquisition (slice thickness <1 mm) and availability of both lung and soft tissue reconstruction kernels. These factors were chosen in order to standardise the chest CT quality in the best way possible, and to exclude external factor causes for nodule detection variability.

Five independent board-certified radiologists (with >10 years of radiological experience each, subspecialising in thoracic radiology [*n* = 3] or paediatric radiology [*n* = 2]) were recruited from different European centres (four United Kingdom, one Netherlands) to independently read the 15 chest CT cases. The readers were asked to detect any lung nodule and provide size measurements during two different sittings (i.e., rounds 1 and 2), 6 months apart.

Readers were blinded to patient symptoms, age and the central radiology review report. The only information provided was that the child had a renal tumour, and that they were to look for and characterise lung nodules on two different occasions. All cases were provided in digital imaging and communications in medicine (DICOM) format and anonymised for the purposes of the study. To minimise recall bias, at the second sitting, DICOM files were re-ordered and renamed with different research identification codes. A blank template completed by each radiologist for each study case was provided (Appendix Table A3) asking readers to state for each nodule the location and size in three dimensions.

To assess the intra- and inter-observer variability for measurement of lung nodules, modified Bland Altman graphs were created to measure inter- and intra-observer limits of agreement (LOA) in size variability for the nodules detected by all five readers, with LOA calculated in all three dimensions.^16–18^

### 2.3 Method III: Simulation study to predict the clinical impact of observer variability

Monte Carlo simulation (MCMC) was performed to examine the clinical consequences when applying the UMBRELLA protocol lung nodule size-staging criteria of ≥3 mm being treated as metastatic disease.^9^ The results from the size distribution (method I) were used to simulate the forthcoming distribution of lung nodules for children enrolled in the UMBRELLA. We predicted a gamma distribution and not exponential distribution, as the smallest nodules are the most difficult to detect, and because we were operating with the CT detected nodules and not the ‘true’ metastases (hence the ones too small to detect).

For the simulation, we used WinBUGS.^10^ Data from the intravariability measurements (method II) were used to simulate the combined effect of the intra- and inter-observer variability of radiologists’ measurements. The model was as follows:

- The per-nodule reference measurement was the mean of all the observers’ measurements.
- The difference between an individual measurement and the reference measurement was assumed to be drawn from a normal distribution.
- The mean of the inter-observer variability was assumed to be the reference measurement.
- The mean of the observations per observer was itself modelled as normally distributed.
- The main observables of the simulation were therefore (per nodule) the variance of measurements per observer and the variance of measurements among observers.

One million MCMC were conducted after a burn-in of 1000. Using the results from above, we then performed the reverse situation, that is, we simulated nodules (*n* = 10,000) using the posterior of nodule size from the MCMC and simulated clinical measurements using the posterior for observer variabilities from the MCMC. Using these data, we then estimated the proportion of nodules within each class (<3 mm vs. ≥3 mm) that may be expected to be misclassified due to observer variability.

## 3 Results

### 3.1 Centralised radiology review

Central radiology review used for this study was performed between January 2014 and October 2017. Chest CT revealed one lung nodule in 84 out of 360 (23%) children with WT, and 42 patients (12%) had more than one lung nodule. The largest sized lesion for each patient ranged from 1 to 80 mm (mean 12 mm).

### 3.2 Assessment of intra- and inter-variability: Radiologists review of chest CT

The subset of 15 chest CTs reviewed originated from eight different UK centres. The mean age of the patients were 4 years 4 months (range: 5 months to 10 years). Three patients (20%) were male.

**Figure 1:**
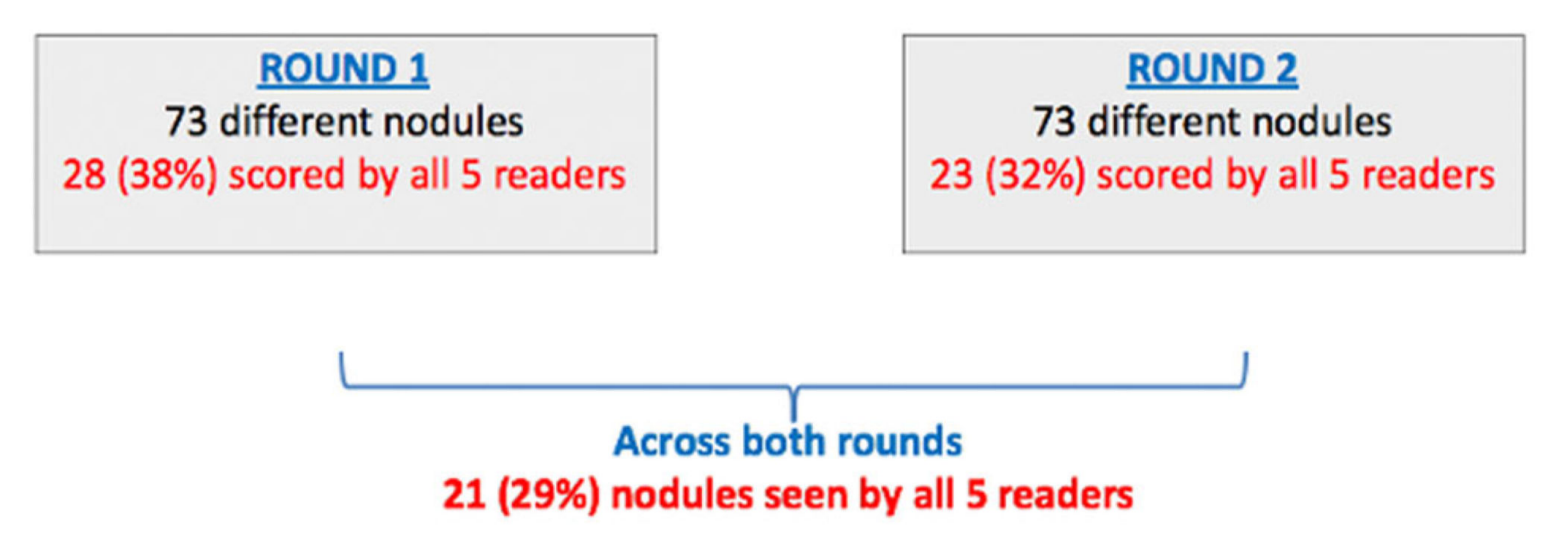
Total number of nodules across both rounds. Overview of the total number of different nodules found across both rounds, and the number of specific nodules found by all five readers. The red numbers are the number of nodules (and in percentage) found by all five readers

In total, 84 different nodules were identified across both rounds (Appendix Table A2). In round 1, 73 different lung nodules were identified, with 28 (38%) of these identified by all five radiologists. In round 2, 73 lung nodules were also identified with 23 (32%) of these identified by all five readers (although these were not all the same 73 nodules in round 1) (Figure 1). In total, 21/73 (29%) lung nodules were identified by all five readers at both rounds of assessment. Intra-variability agreement for nodule detection within each reader for both rounds ranged from identification of 63% to 76% (Table 1).

#### 3.2.1 Variability in size measurement

**Figure 2:**
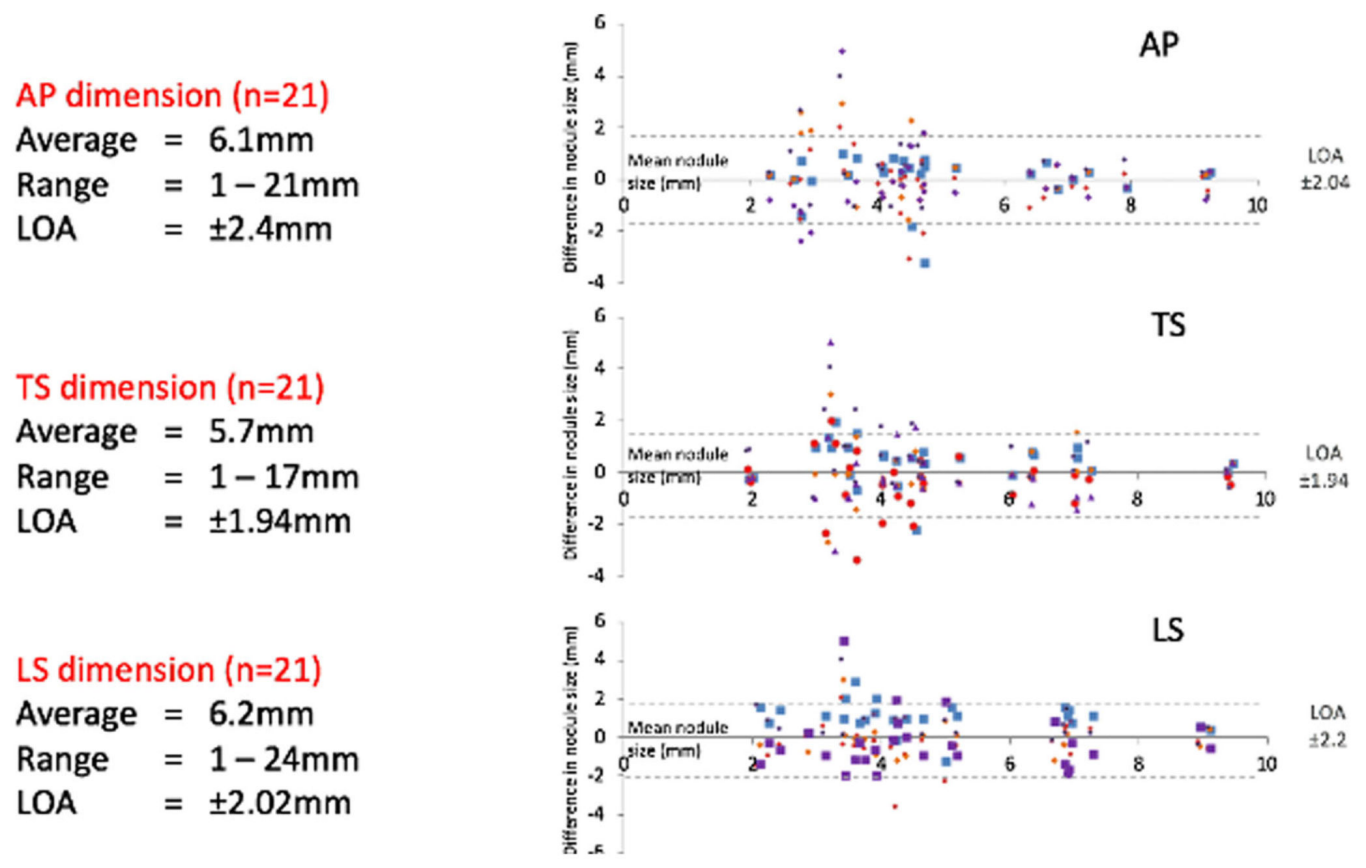
Inter-reader variability limit of agreement (LOA) for round 1. Graph showing the calculated LOA in the three dimensions: AP (antero-posterior),TS (transverse) and LS (cranio-caudal).The LOA shows the difference in nodule size measurement as the deviation from the mean nodule size (0 in difference)

By using the size measurements from the 21 nodules identified by all five readers, inter-observer LOA (in mm) for the five readers were ±2.4 and ±1.4 (antero-posterior diameter), ±1.9 and ±1.8 (transverse diameter) and ±2.0 and ±2.4 (cranio-caudal distance) at assessments 1 and 2, respectively (Figure 2). Intra-observer LOA across the three dimensions were ±1.5, ±2.2, ±3.5, ±3.1 and ±2.6 mm (readers 1–5, respectively).

#### 3.2.2 Clinical impact of observer variability

The UMBRELLA protocol stratifies treatment according to nodule size (i.e., ≥3 mm as metastatic disease). Specifically, for our five expert radiologists, intra-observer variability in size measurements would lead to different treatment allocation for two of 21 (10%) nodules for one reader and in one of 21 (5%) for two other readers (Appendix Figure A1). Likewise, inter-observer variability would lead to different treatment allocation in 14% (3/21) of the cases across both rounds and across all five readers (Table 2).

### 3.3 Simulation study to predict the clinical impact of observer variability

**Figure 3:**
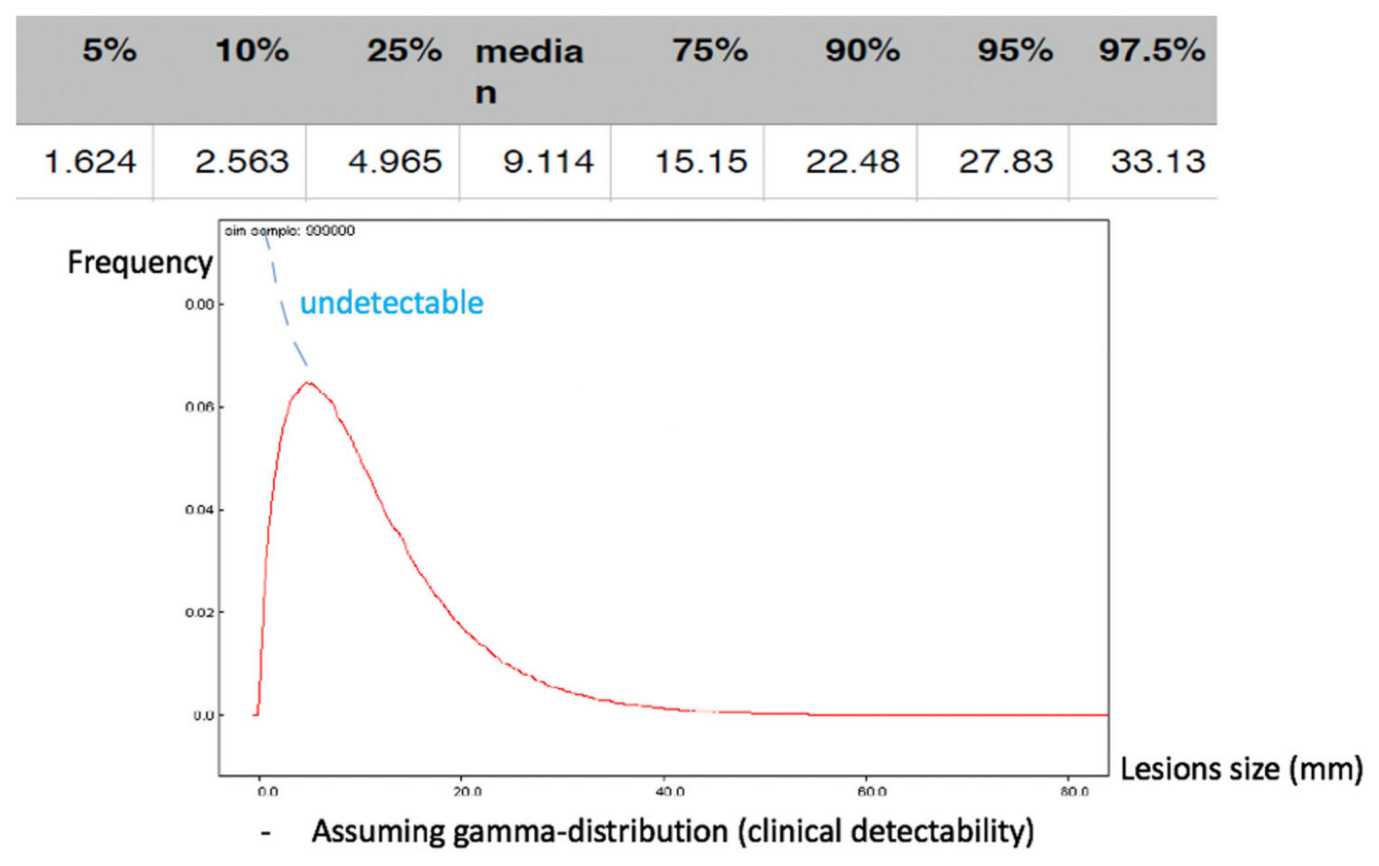
Distribution of lung nodule sizes. The red graph shows the expected size distribution of detectable lung nodules (gamma distribution). The median size of lung nodules is 9 mm. The blue graph shows the indictable lung lesions, hence the ones that are too small to catch on CT (exponential distribution)

Assuming a gamma distribution, a graph displays the expected size distribution of detectable lung nodules (Figure 3). By defining the average measurement of the readers’ observations as a reference, the estimated probability for the deviation of any single observation by any single reader was calculated. It was found that 95% of the measurements are expected to deviate by approximately 4 mm in size (from overestimation of 1.96 mm to underestimation by -1.96 mm) (Appendix Figure A2).

Total 10,000 cases were accumulated in the MCMC to simulate the distribution of proportion of patients with lung nodules of ≥3 or <3 mm. Using the above information, MCMC (*n* = 10,000) demonstrated that in 17% of the patients with a detectable lung lesion and a ‘true’ nodule size of ≥3 mm, the general radiologists across child oncology centres will score the nodule as being <3 mm, thus the patient will be misclassified according to the UMBRELLA protocol and likely be undertreated. In contrast, for 21% of the patients with a detectable lung lesion and a ‘true’ nodule size of <3 mm, the radiologists will score the nodule as being ≥3 mm. Thus, the patients will be misclassified and likely overtreated. Overall, 38% of WT patients with any detectable lung lesion would be allocated to a suboptimal treatment group in our simulation scenario (Table 3).

## 4 Discussion

The inter- and intra-observer variability in this study has shown that size measurements of pulmonary nodules are fragile criteria to determine metastatic disease. As the SIOP-RTSG UMBRELLA protocol for children with WT stratifies treatment according to lung nodule size to define metastatic disease, we have simulated that this variability may have an extensive impact on treatment stratification, and likely clinical outcomes in about one out of three patients with a lung nodule revealed on CT.

The UMBRELLA protocol’s definition of pulmonary metastatic disease is the presence of one or more lung nodule(s) ≥3 mm; patients with metastatic disease would initially receive intensified preoperative treatment with three drugs (actinomycin D, vincristine, doxorubicin [AVD] for 6 weeks) and potentially lung radiotherapy in contrast to patients with localised disease who receive only two drugs (AV for 4 weeks) prior to surgery and no lung radiotherapy.^11^ In the worst case, misclassification and overtreatment with additional doxorubicin and radiotherapy may lead to substantial late effects, whereas misclassification and undertreatment may lead to a higher risk of relapse.^9,19–21^ The presence of small nodules seems associated with an inferior outcome compared to localised disease.^6^ Resection of these small lesions is difficult and thus difficult to obtain histological confirmation of the nature (benign or malignant). This calls for a treatment strategy that does not mandate histological confirmation of small or resolving lung nodules, but one that restricts exposure to additional chemotherapy with a small total dose of doxorubicin and avoids lung RT where small nodules disappear without histological confirmation. While there may be even more variation for the group with the smallest nodules (3–5 mm), the UMBRELLA protocol has added an additional risk group (Appendix Table A1) for this group to further minimise additional treatment to the levels of doxorubicin (150 mg) given by the North American Child Oncology Group (COG) and no radiotherapy if they resolve.

In adults, studies have shown inter- and intra-observer variability for measurement of small pulmonary nodules, respectively, about 1.7 and 1.3 mm.^22^ As nodules in adults are better delineated given the greater surrounding aeration, the measurement variability in children will likely be higher, which our data confirm (inter- and intra-observer variability: 1.4–2.4 and 1.5–3.5 mm, respectively). Furthermore, identification of small lung nodules on chest CT is a common finding in otherwise healthy children.^23^ Therefore, distinguishing benign from malignant lung nodules in children with malignant solid tumours is a real challenge considering the high prevalence of benign lesions and the considerable observer variability.^24^ This was verified by a retrospective study that showed a substantial disagreement (benign vs. malignant) between biopsy results of lung nodules and the initial interpretation of the CT chest.^24^ A similar study showed a variety of patterns on CT in children with lung nodules, which concluded that none of the nodule features studied on CT, reliably differentiated benignity from malignancy when compared with biopsy of the studied lung nodules.^25^ Hence, better technique and/or predictors of metastatic disease are needed. In this vein, a recent study, although still with moderate inter-reader agreement, demonstrated that the axial 5 mm maximum intensity projection techniques used in conjunction with 1-mm slices improve the detection of pulmonary nodules in chest CTs of children and that double reading seems to increase diagnostic reliability in chest CTs of children with a malignancy.^26^ Additionally, computer-based automated volumetry nodule volume measurements seem better than manual unidimensional measurements to characterise lesions, and finally that expert validated structured interventional harmonisation assessment guidelines may improve inter-rater agreement.^24,25,27–30^

Even if radiologists perfectly agree on lung nodule detection and measurements, this does not translate into a clear-cut optimal treatment strategy. The UMBRELLA protocol’s new threshold, defining metastatic disease, captures a group of patients with lung nodules in a *grey* zone. Such patients have previously been labelled as ‘CT-only’ (typically 3-10 mm), as their detection on chest radiographs (CXR) was not easily detected.^14,15^ These CT-only patients seemed to have inferior outcomes compared to the localised disease group. However, the impact of more intensive treatment (three vs. two drugs) for this CT-only group did show a trend, though not a convincing difference in relapse-free survival or overall survival in retrospective cohorts from COG and SIOP.^9,20^ A randomised control trial may be needed to establish the benefits and harms of three versus two drugs for this subgroup.

Despite several attempts, it has been difficult to provide a clear-cut definition of metastatic lung nodules taking underlying disease, size, characteristics and numbers of nodules on chest CTs into account.^31^ Therefore, the UMBRELLA protocol mandates adequate ‘diagnostic quality’ CT imaging studies and a central radiology for all patients to avoid single assessor interpretation. Furthermore, all data should be collected in a library to learn, increase familiarity and to gain more knowledge. The use of artificial intelligence to interpret CT studies has been attempted with the aim of measuring and characterising lung nodules more precisely than humans, which shows promising results.^31,28,32^

### 4.1 Limitations and strengths

Our selection of cases for inter- and intra-observer variability was biased towards imaging of high-quality and reduced motion artefact. In reality, imaging may be of poorer quality and therefore inter-reader agreement, already modest in our study, may in fact be worse in practice. Second, our readers were aware that this study focused on the detection of lung nodules. This may have influenced over-calling of certain abnormalities, which in reality they would otherwise have ignored. Other would argue that in regular practice, radiologists are likely to exercise heightened vigilance in detecting such nodules when aware of the presence of a tumour, so the effect of over-calling in our study is probably limited. The presence of additional clinical information from multidisciplinary team meetings, for example, lung symptoms (which the observers did not have for this study) may influence overall assessments in a real-world clinical setting. Third, we only assessed the largest nodules visible by all readers from the CTs. Hence, smaller lesions not identified by all readers would likely have accounted for an increased variability and overlooking smaller nodules is also a concern. Finally, our MCMC was a simulation using certain assumptions to predict outcomes, and it does not perfectly mirror reality, but provides an approximation and estimate of the clinical impact we expect to witness in the clinic due to intra-inter variability.

## 5 Conclusion

Our study has demonstrated that about one in four children with WT have lung nodules on chest CT at diagnosis. If a threshold of ≥3 mm is used for a lung nodule to dictate metastatic status and intensified treatment, as per the SIOP-RTSG UMBRELLA protocol, radiology observer variability may lead to imprecise risk classification in a substantial proportion of patients. Computer-aided diagnostics or central radiology review with structured assessment could be methods to minimise variability and optimise treatment decision in WT patients with lung nodules revealed on CT. Furthermore, there is an essential need for libraries with data on chest CT scans to learn and improve classification of truly metastatic WT. Such initiatives are implemented in the UMBRELLA protocol.

## Supplementary Material

Appendix

## Figures and Tables

**Table 1:** Intra-variability in number of nodules

Reader	Round 1 Nodules (*n*)	Round 2 Nodules (*n*)	Agreement in both rounds (in %)
1	41	39	34 (74%)
2	54	51	43 (69%)
3	49	41	39 (76%)
4	54	64	48 (69%)
5	39	39	30 (63%)

**Table 2:** Inter-intra variability of lung nodules, size measurement

Nodule number	Reader 1 (mm)		Reader 2 (mm)		Reader 3 (mm)		Reader 4 (mm)		Reader 5 (mm)	
	Round 1	Round 2	Round 1	Round 2	Round 1	Round 2	Round 1	Round 2	Round 1	Round 2
1	7	7	5	6	5	6	6	5	6	5
2	5	5	4	3	4	3	4	4	3	3
3	4	5	3	2	7	7	7	7	7	7
4	6	5	5	5	4	3	6	5	6	5
5	6	5	3	4	4	4	6	5	5	5
6	6	6	5	5	7	6	7	7	2	6
7	4	5	4	3	3	3	4	4	4	3
8	4	4	3	2	2	3	3	3	2	2
9	8	8	8	7	8	7	8	8	8	7
10	9	8	7	7	7	6	8	7	7	6
11	6	6	5	5	6	3	4	3	3	4
12	5	5	5	5	5	5	6	5	6	5
13	5	4	4	4	3	4	7	6	5	4
14	4	4	3	3	3	4	3	4	4	4
15	10	10	10	9	9	9	9	10	9	10
16	6	5	5	5	6	6	6	5	5	5
17	7	8	7	7	7	7	8	8	6	6
18	5	5	5	4	3	4	4	5	5	4
19	14	12	12	12	12	12	13	12	13	13
20	23	22	21	21	10	19	22	18	22	24
21	10	12	12	11	12	11	11	12	12	14

**Table 3:** Observer variability and treatment intensity

Observed diameter	Actual/’true’ diameter	Frequency with impact on staging
<3 mm	≥3 mm	17% (downstage to AV)
≥3 mm	<3 mm	21% (upstage to AVD)

Abbreviations: AV, actinomycin D, vincristine; AVD, actinomycin D, vincristine, doxorubicin.

## Data Availability

Data are available via the authors at a local database if required or needed.

## Abbrevations

CXR: chestX-ray
DICOM: digital imaging and communications in medicine
IMPORT: Improving Population Outcomes of Renal Tumours of childhood study
LOA: limits of agreements
MCMC: Monte Carlo simulation
SIOP-RTSG: Société Internationale d’Oncologie Pédiatrique Renal Tumour Study Group
WT: Wilms tumour
