# Supplementary material for: The clinical impact of observer variability in lung nodule classification in children with Wilms tumour
Source: Pediatr Blood Cancer. Author manuscript; Available in PMC 2023 Oct 12. (PMC7615195; doi:10.1002/pbc.29759)
Supplement: Appendix [file EMS188583-supplement-Appendix.docx]

**Appendix**

**Supplemental table 1. Stage IV treatment overview from the SIOP umbrella protocol 2016**

| Overall Res-ponse | Metastasis  Surgery | Nephro-blastoma histology | Treatment | |
| --- | --- | --- | --- | --- |
| CR/ VGPR | Surgical complete resection if needed | LR/IR & lung nodules 3-5 mm | AVD150, no pulmonary RT unless complete resection of still viable metastasis 🡪 pulmonary RT | |
|  |  | LR/IR & lung nodules >5 mm or other site | AVD250, no pulmonary RT unless complete resection of viable metastasis 🡪 pulmonary RT | |
|  |  | LR/IR | No evidence of viable tumour | Treatment as localized (Chapter 15.2) |
| PR/SD | Representative nodule resection feasible | LR | Viable metastasis confirmed | AVD250, lung/metastasis RT, CT at week 10 🡪 if remaining nodules 🡪 surgery recommended to achieve CR if feasible |
|  |  |  | Completely necrotic metastasis | AVD150, CT at week 10 🡪 if remaining nodules 🡪 surgery recommended to achieve CR if feasible |
|  |  | LR/IR | No evidence of viable or necrotic metastasis | Contact principal investigator potentially treatment as localized (Chapter 15.2) or AVD250, CT week 10 🡪 if remaining nodules 🡪 surgery recommended to achieve CR if feasible, no RT to metastatic site(s) |
|  |  | IR | Viable metastasis confirmed | 4 drug regimen, RT to metastasis. CT week 10 🡪 if remaining nodules 🡪 surgery recommended to achieve CR if feasible |
|  |  |  | Completely necrotic metastasis | AVD250 regimen, CT week 10 🡪 if remaining nodules 🡪 surgery recommended to achieve CR if feasible |
|  | Resection not feasible | LR | AVD250, CT week 10 🡪 remaining nodules reconsider resection and discuss RT to metastasis | |
|  |  | IR | 4 drug regimen, CT week 10 🡪 if remaining nodules RT to metastasis indicated | |
| PD | Representative nodule resection feasible | IR | Metastasis confirmed | 4 drug regimen, metastasis RT. CT week 10 🡪 if remaining nodules 🡪 surgery recommended to achieve CR if feasible |
|  |  |  | No evidence of viable or necrotic tumour | AVD250, CT week 10 🡪 if remaining nodules 🡪 surgery 🡪 if viable metastasis 🡪 CDCV + RT to metastases indicated: contact PI (these situations will be very rare). |
| All | All | HR | Ask PI for advice, metastasis RT, CT week 10 🡪 if remaining nodules consider resection if feasible | |
| Mixed | Indicated | Confirm metastatic disease by histology 🡪 if metastasis 🡪 treat according to worst histology and worst response | | |

**Supplemental table 2. Number of nodules for all 5 readers, round 1 and 2**


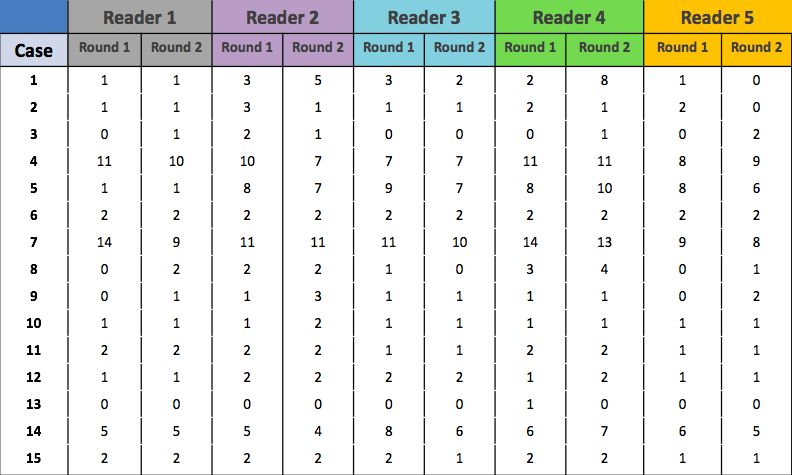


**Supplemental figure 1: Pulmonary nodules reported by all five readers**

*
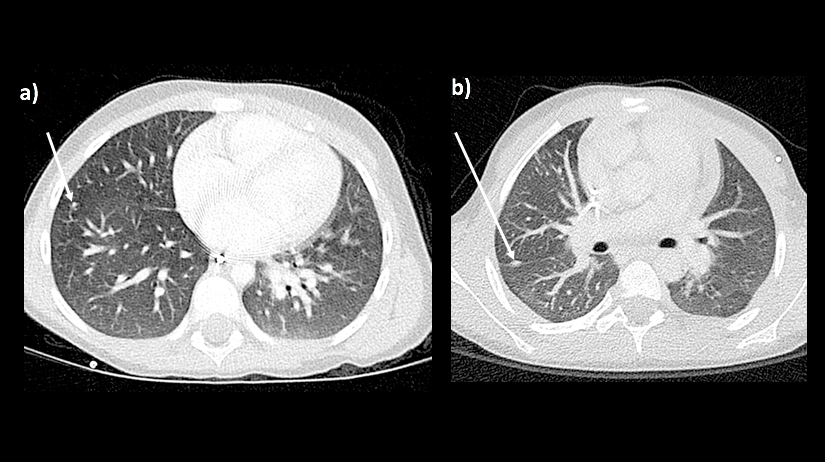
*

*Two examples of pulmonary nodules reported by all five readers, but where the limits of agreement in nodule measurement between radiologists would change the treatment stratification .*

1. *A right lower lobe nodule (white arrow) in a 4 year old female patient (corresponding to nodule 8, Table 2)*
2. *A right perifissural nodule along the right oblique fissure in a 6 year old female patient (corresponding to nodule 6, Table 2)*

**Supplemental figure 2. Average size variability**


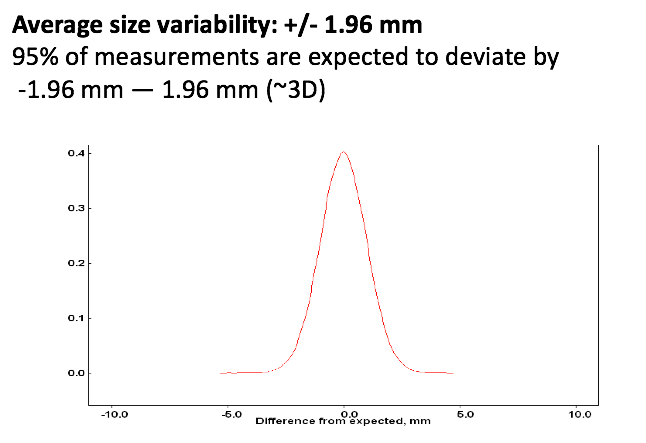


**Supplemental table 3. Pulmonary Nodules in Wilms Tumors Scoring Sheet**

CASE NUMBER: _______________________

| Nodule Number | 1 | 2 | 3 | 4 | 5 | 6 | 7 | 8 |
| --- | --- | --- | --- | --- | --- | --- | --- | --- |
| Nodule Location  (RUL, RML, RLL, LUL, LLL) |  |  |  |  |  |  |  |  |
| Nodule Location within lobe  (central, peripheral, perifissural) |  |  |  |  |  |  |  |  |
| Slice Number (Axial, Thin Slices) |  |  |  |  |  |  |  |  |
| Maximal AP distance (mm) |  |  |  |  |  |  |  |  |
| Maximal TS distance (mm) |  |  |  |  |  |  |  |  |
| Maximal LS distance (mm) |  |  |  |  |  |  |  |  |
| Smooth Margins? (Y/N) |  |  |  |  |  |  |  |  |
| Calcification? (Y/N) |  |  |  |  |  |  |  |  |
| Characteristic  (solid, subsolid, ground glass) |  |  |  |  |  |  |  |  |
| Nodule Shape  (polygonal, round, oval, complex) |  |  |  |  |  |  |  |  |
| Impression  (benign, malignant, indeterminate) |  |  |  |  |  |  |  |  |
| Confidence of Impression  Scale of 1 to 10  (10= completely certain, no doubts) |  |  |  |  |  |  |  |  |

Please tick here if you do not think there are any nodules within the imaging at all
